# Regulating BRCA1 protein stability by cathepsin S-mediated ubiquitin degradation

**DOI:** 10.1038/s41418-018-0153-0

**Published:** 2018-07-13

**Authors:** SeoYoung Kim, Hee Jin, Hang-Rhan Seo, Hae June Lee, Yun-Sil Lee

**Affiliations:** 1 Graduate School of Pharmaceutical Sciences, Ewha Womans University, Seoul, 120-750 Korea; 2 Functional Morphometry II, Institute Pasteur Korea, Bundang-gu, Seongnam-si, Gyeonggi-do 463-400 Korea; 3 Division of Basic Radiation Bioscience, Korea Institute of Radiological and Medical Sciences, Seoul, 139-706 Korea

**Keywords:** Cancer models, Tumour-suppressor proteins, Tumour-suppressor proteins, Cancer models, Tumour-suppressor proteins

## Abstract

Cathepsin S (CTSS) is a cysteine protease that is thought to play a role in many physiological and pathological processes including tumor growth, angiogenesis, and metastasis; it has been identified as a radiation response gene. Here, we examined the role of CTSS in regulating the DNA damage response in breast cancer cells. Activating CTSS (producing the cleavage form of the protein) by radiation induced proteolytic degradation of BRCA1, which ultimately suppressed DNA double-strand break repair activity. Depletion of CTSS by RNAi or expression of a mutant type of CTSS enhanced the protein stability of BRCA1 by inhibiting its ubiquitination. CTSS interacted with the BRCT domain of BRCA1 and facilitated ubiquitin-mediated proteolytic degradation of BRCA1, which was tightly associated with decreased BRCA1-mediated DNA repair activity. Treatment with a pharmacological CTSS inhibitor inhibited proteolytic degradation of BRCA1 and restored BRCA1 function. Depletion of CTSS by shRNA delayed tumor growth in a xenograft mouse model, only in the presence of functional BRCA1. Spontaneously uced rat mammary tumors and human breast cancer tissues with high levels of CTSS expression showed low BRCA1 expression. From these data, we suggest that CTSS inhibition is a good strategy for functional restoration of BRCA1 in breast cancers with reduced BRCA1 protein stability.

## Introduction

BRCA1 (breast cancer susceptibility gene 1), a tumor suppressor, participates in DNA double-strand break (DSB) repair, S and G2/M cell cycle checkpoints after damage, control of centrosome number, maintenance of heterochromatin, and transcriptional regulation of several genes [1–3]. BRCA1 dysfunction in tumors is known to lead to impaired homologous recombination (HR)-mediated DSB repair, resulting in significant genomic instability. Recruitment of BRCA1 to DNA DSBs facilitates repair by HR, whereas loss of BRCA1 results in genomic instability characterized by unrepaired DNA breaks and complex chromosomal rearrangements that compromise cell viability [4–6]. Thus, BRCA1 is emerging as a central mediator of the cellular mechanism that maintains genome stability by bringing together multiple signaling complexes in response to DNA damage. Although BRCA1 mutations account for a significant proportion of familial breast and ovarian cancers, reduced BRCA1 protein is also reported in sporadic breast cancers, and expression of BRCA1 protein is frequently lost in breast cancer patients; this loss of expression is associated with disruption of critical functions in cells and with cancer development. The mechanisms of BRCA1 silencing in sporadic breast cancer cells are known to cover the full spectrum of genetic and epigenetic mechanisms, including somatic mutations, transcriptional repression, microRNA-based down-regulation or translational blockade, and generation of alternately spliced variants [7]. However, regulation of BRCA1 protein stability is not fully understood. BRCA1 is ubiquitinated and degraded during tumorigenesis [8], and BRCA1 protein level is also regulated in a cell cycle-dependent manner [9]. One significant factor that regulates the stability of BRCA1 is the protein BARD1, which associates with BRCA1 to form a RING heterodimer that is essential for BRCA1 stability, nuclear localization, and E3 ligase activity [10]. Another mechanism of BRCA1 degradation is HERC2-mediated BRCA1 ubiquitination. HERC2 also interacts with the RING domain of BRCA1 and regulates BRCA1 stability in opposition to BARD1 [11]. The Skp1-Cul1-F-box-protein44 (SCFFBXO44) complex is also reported to ubiquitinate full-length BRCA1. Furthermore, the RING domain of BRCA1 mediates the interaction between BRCA1 and FBXO44. Over-expression of SCFFBXO44 reduces the BRCA1 protein level [12].

BRCA1 encodes a polypeptide of 1,863 amino acids that contains an N-terminal RING domain and tandem C-terminal BRCT domains. The RING domain of BRCA1 has E3 ubiquitin ligase activity [13–23], whereas the BRCT domain has been demonstrated to be a phospho-protein interaction domain [24–27]. Observations that the BRCT domains are frequently targeted by many clinically important mutations and that most of these mutations disrupt the binding surface of the BRCT domains to phosphorylated peptides indicate that the BRCT domains are integral for the tumor suppressor function of BRCA1 [28]. Indeed, the BRCT domain of BRCA1 has been shown to be important for cell cycle checkpoint, HR, and tumor suppression [29–33].

The cathepsin family of cysteine proteases has been implicated in processes that are important for tumor development and progression [34], and increased levels of cathepsin have been detected in cancer [35, 36]. In addition to these observations, there is now ample clinical evidence that up-regulation of these proteinases confers a poor prognosis in patients with a variety of malignancies [37–39]. The human cysteine cathepsin family comprises 11 genes that encode intracellular proteases that are crucially important for terminal protein degradation in the acidic environment of lysosomes [40]. Cathepsisn S (CTSS) is distinguished from other cysteine proteinases by its limited tissue distribution and better conformational stability at neutral and slightly basic pH. It is suggested that CTSS is often overexpressed in cancerous tissues and cells, with the highest level of CTSS activity in the most malignant tumors [41]. These observations provide evidence supporting a potential role of CTSS in cancer development, although more work is needed to confirm such a function.

In our previous study, we identified CTSS as a gene that is up-regulated specifically in ionizing radiation (IR)-induced rat mammary tumors and that showed that over-expression of CTSS was involved in cellular transformation [42]. Moreover, IR-induced cytokines including interferons, tumor necrosis factors, and interleukins are potent regulators of CTSS expression via indirect pathways such as reactive oxygen species production [43]. In this study, we further elucidated the molecular mechanisms of CTSS-mediated tumorigenesis, showing that CTSS affects a DNA damage response protein, BRCA1. CTSS interacted with BRCA1 and cleaved its BRCT domain, which facilitated ubiquitin-mediated degradation of BRCA1 and ultimately resulted in a decreased DNA damage response and defective repair activity. Moreover, pharmacological inhibition of CTSS restored BRCA1 stability, which correlated with enhanced BRCA1 function.

## Results

### CTSS inhibited BRCA1 expression

**Fig. 1:**
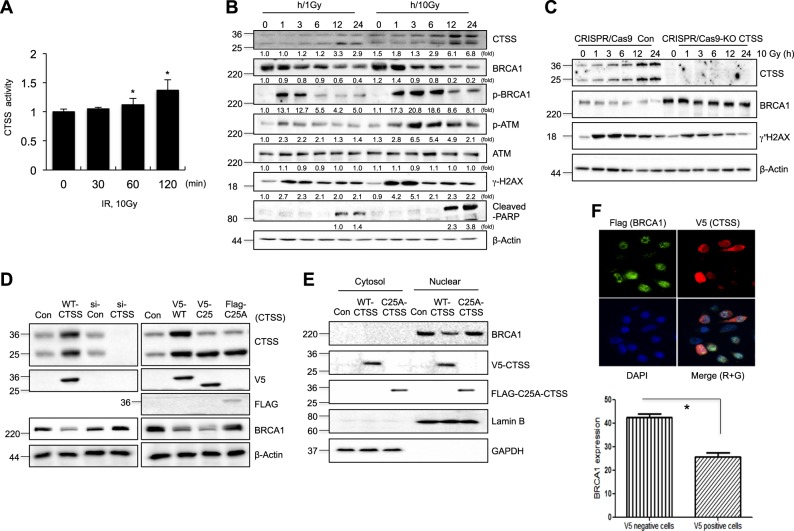
CTSS modulates BRCA1 protein expression. **a** CTSS protease activity was detected using MCF7 cells after exposure to 10 Gy (IR) at indicated time points. *p < 0.05 vs. untreated control cells (Student’s *t*-test). **b** MCF7 cells were irradiated with 1 Gy and 10 Gy and after indicated time points, Western blotting was performed on the cells. Protein levels were quantified using Image J software, and data are expressed as the fold change relative to the negative control. **c** Western blot analysis of CRISPR-Cas9-Control (Con) and -CTSS KO MCF7 cells in the presence or absence of 10 Gy radiation. **d** MCF7 cells transfected with WT-CTSS, si-RNA, or mutant type of CTSS (C25A; active site Cys25 mutated to Ala). **e** Western blotting of cytosolic and nuclear fractions from MCF7 cells was performed. Fraction purity and equal loading were assessed by Western blots for Lamin B and GAPDH. **f** MCF7 cells were irradiated (10 Gy) for 2 h and stained with Flag antibody for BRCA1 (green, **g**), V5 antibody for CTSS (red, R), and DAPI. Cells were analyzed using immunofluorescence microscopy. Quantification of BRCA1 staining was performed by dividing into V5 positive and negative MCF7 cells. The error bars represent S.D. Data are expressed as the fold change relative to the control. *p < 0.05 (Student’s *t*-test)

A previous study suggested that IR or H_2_O_2_ activated CTSS [43]. CTSS activity increased with 60 min of IR (Fig. 1a), and IR resulted in dose dependent cleavage of inactive CTSS precursor (36 kDa) into its activated form (24 kDa), which is capable of degrading a range of macromolecules in MCF7 cells (Fig. 1b, c). Screening of CTSS targets for DNA damage response proteins indicated that BRCA1 expression was reduced in CTSS over-expressing cells but that other proteins such as ATM, DNA-Pkcs, Ku80, and Cyclin D1 were not affected (Supplementary Figure S1A). Decreased BRCA1 stability was induced from 3 h of 10 Gy IR, which coincided with initiation of CTSS cleavage. BRCA1 phosphorylation was occurred before completion of BRCA1 degradation, and ATM phosphorylation increased continuously and it lasted until 12 h of IR. The peak induction of γH2AX was occurred at 1 and 3 h of 10 Gy IR and it lasted up to 24 h. Cell death marker PARP1 was induced at 12 and 24 h of 10 Gy IR. In the case of low dose of 1 Gy IR, these effects were weaker than 10 Gy irradiated cells (Fig. 1b). The active form of CTSS (C25-CTSS) showed stronger reduction of BRCA1 level than the wild-type precursor form of CTSS (WT-CTSS), whereas knockdown of CTSS by siRNA (si-CTSS) or CRISPR/CAS9-KO system had the opposite effect and increased the BRCA level. Moreover, mutation of the cysteine residue at amino acid 25 (C25A-CTSS), which inactivates the CTSS protease activity, did not inhibit BRCA1 expression, and a CTSS-specific small molecule inhibitor VBY-036 (VBY) restored BRCA1 expression (Fig. 1c, d, and Supplementary Figures S1B and S1C). Because BRCA1 is usually located in the nucleus, we examined cellular localization after CTSS expression. Western blot analysis of cellular fractions showed that CTSS inhibited BRCA1 expression in the nucleus, whereas the mutant C25A form of CTSS did not (Fig. 1e). Immunofluorescence data also indicated that V5-CTSS positive cells showed low expression of BRCA1 foci after IR than non-transfected negative cells (Fig. 1f). siRNA silencing of other cathepsins such as cathepsin L (CTSL) or cathepsin B (CTSB) did not result in inhibition of BRCA1 expression, and a pan inhibitor of cathepsin that can inhibit all cysteine cathepsins (E-64) did not fully restore BRCA1 expression when used at the same concentration as the CTSS specific inhibitor, VBY (Supplementary Figure S1C), suggesting that CTSS was activated by DNA damage response and specifically inhibited BRCA1 expression.

### BRCT domain of BRCA1 was cleaved by CTSS

**Fig. 2:**
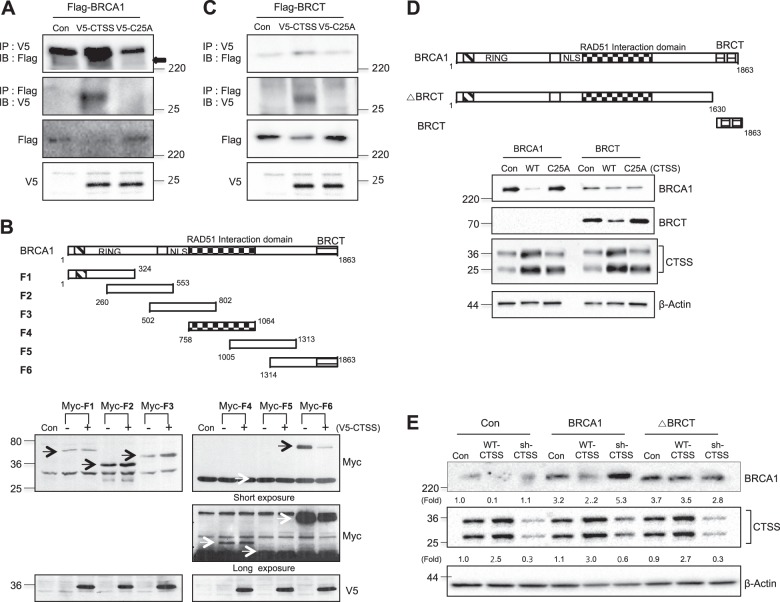
CTSS interacts with the BRCT domain of BRCA1 and inhibits BRCA1 protein expression. **a** Constructs of V5-CTSS and V5-C25A (active site of Cys25 mutated to Ala) were transiently transfected into Flag-BRCA1 transfected MCF7 cells, and cell extracts were subjected to immunoprecipitation (IP) and immunoblotting (IB). **b** Schematic presentation of BRCA1 domain, including RING, Rad51 interacting, and BRCT domains (upper). MCF7 cells were transfected with BRCA1 fragments (F1 to F6) encoding Myc with or without full-length V5-CTSS and analyzed by Western blotting (bottom). **c** Constructs of V5-CTSS and V5-C25A were transiently transfected into Flag-BRCT transfected MCF7 cells, and cell extracts were subjected to immunoprecipitation (IP) and immunoblotting (IB). **d** Schematic structure of BRCA1, BRCT, and BRCT deletion construct (ΔBRCT). MCF7 cells were transfected with WT-BRCA, BRCT with and without WT-CTSS, C25A and analyzed by Western blotting. **e** MCF7 cells were transfected with WT-BRCA and ΔBRCT constructs with and without WT-CTSS or sh-CTSS and analyzed by Western blotting. Protein levels were quantified using Image J software, and data are expressed as the fold change relative to the negative control

Because mRNA expression of BRCA1 was not altered by CTSS over-expression (Supplementary Figure S1D), we hypothesized that CTSS cleaves BRCA1 and thus regulates the protein level. Using immunoprecipitation (IP), we found that WT-CTSS interacted with BRCA1 but C25A did not (Fig. 2a). IP with deletion constructs of BRCA1 indicated that CTSS regulated expression of the F6 domain of BRCA1 that includes the BRCT domain but not other domains (Fig. 2b). Indeed, IP analysis confirmed that CTSS interacted with the BRCT domain of BRCA1 (Fig. 2c). For more detailed experiments, we prepared BRCT domain only and BRCT domain-deleted BRCA1 mutant form (ΔBRCT). BRCT expression decreased with WT-CTSS over-expression but not with the C25A mutant form of CTSS (Fig. 2d). Moreover, when ΔBRCT was transfected into the cells, BRCA1 expression was not altered by CTSS (Fig. 2e), suggesting that CTSS interacts with and cleaves the BRCT domain of BRCA1 and regulates protein expression.

### CTSS regulated ubiquitin-mediated degradation of BRCA1

**Fig. 3:**
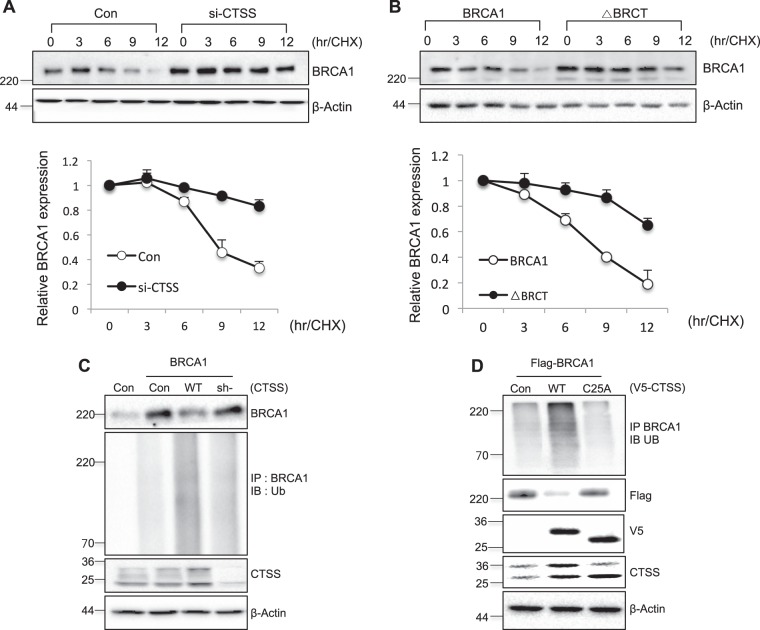
CTSS increases ubiquitin-mediated degradation of BRCA1. **a** Western blot analysis in control or si-CTSS transfected MCF7 cells treated with 100 μg/ml cycloheximide (CHX) for various lengths of time. **b** MCF7 cells were transfected with WT-BRCA1 or BRCT deletion mutant of BRCA1 (ΔBRCT) and incubated in the presence of CHX, and Western blotting was performed. Band density was expressed as the fold change relative to the control (Mean ± SD of 3 experiments). **c**, **d** For ubiquitination assays, MCF7 cells were transfected with control, WT-CTSS, C25A-CTSS, sh-CTSS, or BRCA1 constructs after transfection with ubiqutine construct (Ub). Cell lysates were immunoprecipitated (IP) and immunoblotted (IB)

To elucidate whether BRCA1 degradation was regulated by CTSS, we treated cells with CHX and examined its stability of BRCA1. Transfection with si-CTSS increased BRCA1 stability (Fig. 3a). The stability of the ΔBRCT also increased compared with WT-BRCA1 (Fig. 3b), suggesting that CTSS cleaved the BRCT domain of BRCA1, which decreased BRCA1’s stability. Because BRCA1 stability was reported to be regulated by ubiquitin proteolysis [44], we examined the effect of CTSS on ubiquitination of BRCA1. We observed BRCA1 ubiquitination in the presence of WT-CTSS but not transfection with sh-CTSS or the mutant form of CTSS (C25A; Fig. 3c, d), suggesting ubiquitin-mediated degradation of BRCA1 by CTSS.

### CTSS regulated BRCA1 functions

**Fig. 4:**
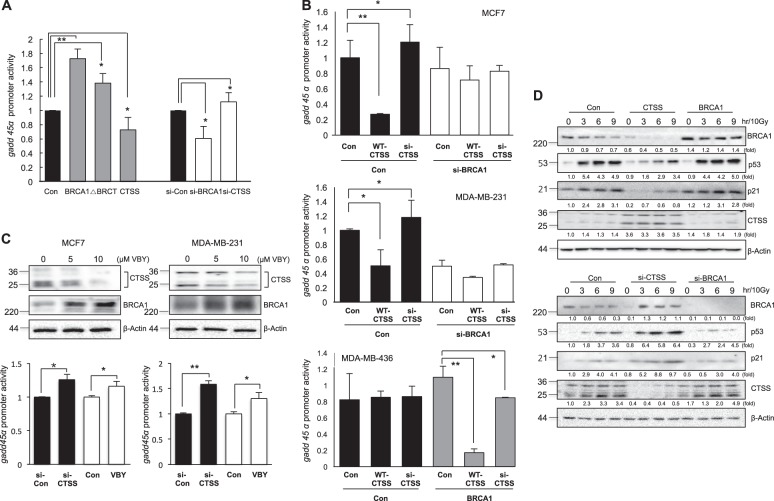
CTSS enhances BRCA1 downstream functions. **a** Promoter reporter construct *gadd*45 was co-transfected with BRCA1, ΔBRCT, CTSS, si-Con, si-BRCA1, or si-CTSS into MCF7 cells. Cells were collected and subjected to luciferase assay. **b** MCF7 (upper), MDA-MB-231 (middle) and MDA-MB-436 (bottom) cells were co-transfected with WT-CTSS or si-CTSS either the si-Con or si-BRCA1 as indicated. **c** CTSS and BRCA1 protein expression following treatment of MCF7 or MDA-MB-231 cells with CTSS specific inhibitor VBY-036 (10 μM) (upper). Promoter activity *gadd*45 was following treatment of MCF7 or MDA-MB-231 cells with CTSS-specific inhibitor VBY-036 or si-CTSS (bottom). **d** MCF7 cells were treated with IR (10 Gy) after transfection with WT-BRCA1, WT-CTSS, si-CTSS, or si-BRCA1 constructs and Western blotting was performed. Protein levels were quantified using Image J software and data are expressed as the fold change relative to the negative control. Normalized luciferase activities were referred to the activity of extracts. Graphs represent Mean ± SD of three experiments. *p < 0.05 and **p < 0.01 (ANOVA)

To elucidate whether CTSS affects BRCA1 functions, we examined the promoter activity of *gadd45*, which is regulated by BRCA1 [45]. We observed increased *gadd45* promoter activity observed in WT-BRCA1 or si-CTSS over-expressing cells, whereas WT-CTSS and si-BRCA1 significantly decreased *gadd45* promoter activity. In the case of ΔBRCT, we observed partial inhibition of *gadd45* promoter activity (Fig. 4a). We did not observe CTSS-dependent alteration of *gadd45* promoter activity in si-BRCA1-treated MCF7 or MDA-MB-231 cells (both cells showed wild-type BRCA1 function and relatively high BRCA1 expression). In the case of BRCT domain-mutated MDA-MB-436 cells, which showed low BRCA1 expression, CTSS did not affect BRCA1expression levels. However, when MDA-MB-436 cells were over-expressed to WT-BRCA1, we did observe CTSS-mediated degradation of BRCA1 (Fig. 4b). The CTSS-specific inhibitor VBY increased *gadd45* promoter activity accompanied by increased BRCA1 expression in MCF7 and MDA-MB-231 cells (Fig. 4c). After IR, the expression of DNA damage response proteins such as p53 and p21, which are reported to be regulated by BRCA1 [9], was also affected by CTSS expression patterns and correlated with the BRCA1 expression levels (Fig. 4d).

### RING domain of BRCA1 was not important for CTSS-mediated degradation of BRCA1

**Fig. 5:**
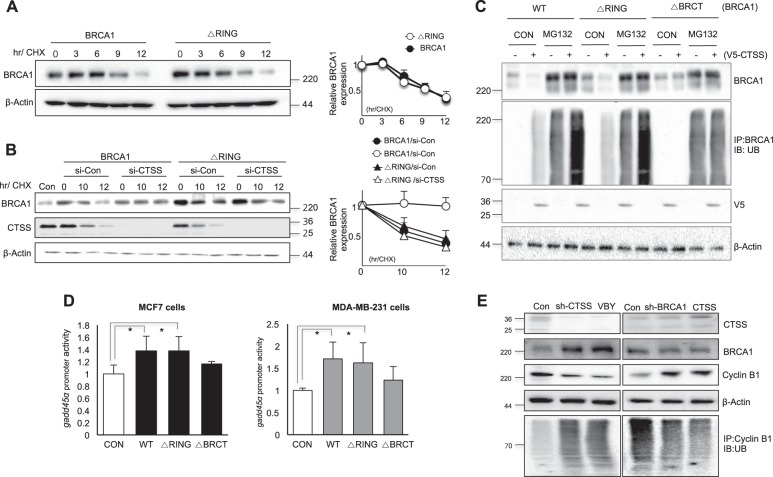
RING domain of BRCA1 is not important for CTSS-mediated BRCA1 degradation. **a** Western blot analysis in WT-BRCA1 and RING domain deleted mutant (ΔRING) transfected MCF7 cells treated with 100 μg/ml cycloheximide (CHX) for various lengths of time. **b** MCF7 cells were transfected with WT-BRCA1 or ΔRING with or without siRNA of CTSS (si-CTSS) and incubated in the presence of CHX and Western blotting was performed. Band density was expressed as the fold change relative to the control (Mean ± SD of 3 experiments). **c** MCF7 cells were transfected with WT-BRCA1, ΔRING, or ΔBRCT. For ubiquitination assays, MG132 (10 μM) was treated after transfection of WT-CTSS, and cell lysates were immunoprecipitated (IP) and immunoblotted (IB) with ubiqutin construct (Ub). **d** Promoter reporter construct *gadd*45 in MCF7 (left) and MDA-MB-231 (right) cells were co-transfected with WT-BRCA1, ΔRING, or ΔBRCT as indicated. Normalized luciferase activity referred to the activity of the extracts. Data are representative of three independent experiments with similar results. Graphs represent mean ± SD of three experiments. *p < *0.05* (ANOVA). **e** Cell lysates after transfection of sh-CTSS or treatment of VBY-036, a CTSS specific inhibitor at 10 μM were immunoprecipitated (IP) with cyclin B1 and immunoblotted (IB) with ubiqutin construct (Ub). Western blotting was also performed

Because BRCA1 degradation is mainly regulated by the RING domain of BRCA1, which has E3 ligase function [46], we examined the role of this domain in CTSS-mediated BRCA1 degradation. The RING-domain deletion mutant (ΔRING) showed slightly increased protein stability in CHX chase experiments compared with WT-BRCA1 (Fig. 5a). However, CTSS knockdown affected WT-BRCA1 protein stability but not that of the ΔRING mutant, suggesting that the target of CTSS for BRCA1 degradation is not the RING domain of BRCA1. Moreover, CTSS did not affect ubiquitination of the ΔRING mutant of BRCA1 (Fig. 5b). Treatment with MG132, a proteasome inhibitor, increased the ubiquitin degradation of BRCA1 and WR-CTSS over-expression facilitated these phenomena. However, ΔBRCT transfection did not affect ubiquitin degradation of BRCA1 even though WT-CTSS was over-expressed, whereas the RING domain deleted mutant of BRCA1 (ΔRING) increased BRCA1 ubiquitination by WT-CTSS (Fig. 5c). We observed increased *gadd45* promoter activity when the ΔRING mutant or WT-BRCA1 was transfected into MCF7 and MDA-MB-231 cells, whereas it was not by ΔBRCT transfection (Fig. 5d). We also examined ubiquitination patterns of cyclin B1, which was reported to be ubiquitinated by BRCA1 [47]. After stable transfection with sh-CTSS and treatment with CTSS inhibitor, VBY increased cyclin B1 ubiqutination mediated by increased BRCA1 expression, whereas over-expression of CTSS or sh-BRCA1 transfection reduced cyclin B1 ubiquitination (Fig. 5e).

### CTSS decreased BRCA1-mediated HR repair activity

**Fig. 6:**
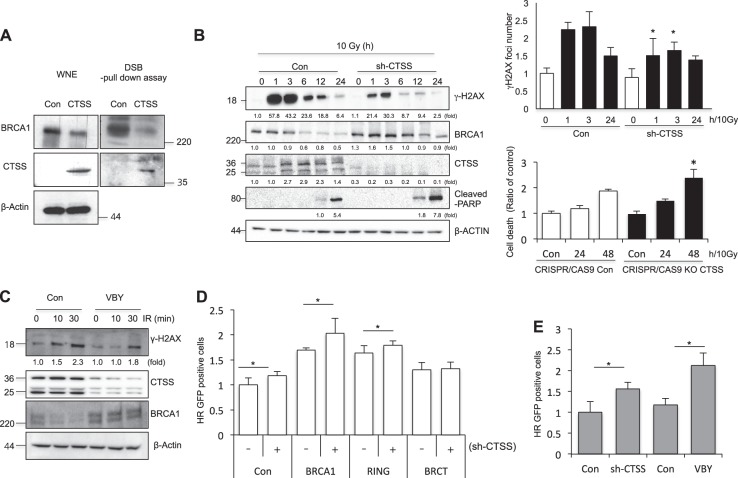
CTSS decreases DNA damage responses. **a** Control or WT-CTSS was transiently transfected into MCF7 cells. The levels of BRCA1 and CTSS in the dsDNA pull-down lysates, as well as in complete whole nuclear extracts, were analyzed. **b** MCF7 cells were irradiated with 10 Gy IR after transfection with control or sh-CTSS, and Western blotting was performed at indicated time points. Protein levels were quantified using Image J software, and data are expressed as the fold change relative to the negative control (left). Immunofluorescence analysis for γH2AX foci were performed after 10 Gy IR (right upper). Apoptotic cells after 10 Gy IR were evaluated by PI staining in CRISPR-Cas9 CTSS KO MCF7 cell lines. *p < *0.01* vs. corresponding control cells (ANOVA) (right bottom). **c** After 10 Gy radiation, Western blotting was performed at indicated time points with or without treatment of CTSS-specific inhibitor VBY-036 (10 μM). **d** The ratio of GFP + cells transfected with sh-CTSS, BRCA1, ΔBRCT, or ΔRING expression in MCF7 cells that stably expressed DR-GFP were analyzed by FACS. **e** GFP + cells after treatment of VBY-036 (10 μM) or sh-CTSS in MCF7 cells that stably expressed DR-GFP were analyzed by FACS (mean ± SD from 3 different experiments). *p < 0.05 (ANOVA)

Because BRCA1 is an important protein for DNA repair pathways, especially HR, we examined whether CTSS affects HR using a DSB pull-down assay with exogenously transfected double-stranded oligonucleotides to evaluate the association of BRCA1 with DNA DSBs. Our data showed that BRCA1 association with DSBs was significantly reduced in CTSS over-expressing cell nuclei compared with that in control cell nuclei (Fig. 6a). Induction of γH2AX and γH2AX foci by IR was decreased in sh-CTSS transfected cells. However, in the case of cell death, it was increased by depletion of CTSS (Fig. 6b and Supplementary Figure S2A). The CTSS inhibitor VBY had similar effects to sh-CTSS treatment (Fig. 6c). Use of reporter systems that can distinguish between the DSB repair pathways NHEJ and HR based on enhanced green fluorescent protein and meganuclease such as I-SceI [48] indicated that CTSS over-expression reduced HR repair activity but not NHER repair activity (Supplementary Figure 2SB). CTSS affected HR repair activity in the RING-domain deletion mutant (ΔRING) but not in the BRCT-deleted mutant (ΔBRCT; Fig. 6d and Supplementary Figure S2C). Treatment with VBY had similar effects to sh-CTSS, with increased HR repair activity (Fig. 6e and Supplementary Figure S2D). We confirmed these data using comet tail moment detection and acquired similar results (Supplementary Figures S3A and S3B). In the case of MDA-MB-436 cells with BRCT domain mutation and low BRCA1 expression, CTSS did not induce any alteration in the comet tail moment in response to IR. However, when we transfected WT-BRCA1 into the MDA-MB-436 cells, CTSS increased the comet tail length induced by IR, whereas si-CTSS inhibited the IR-induced comet tail length (Supplementary Figure S3C).

### CTSS inhibition increased IR-mediated cell death

**Fig. 7:**
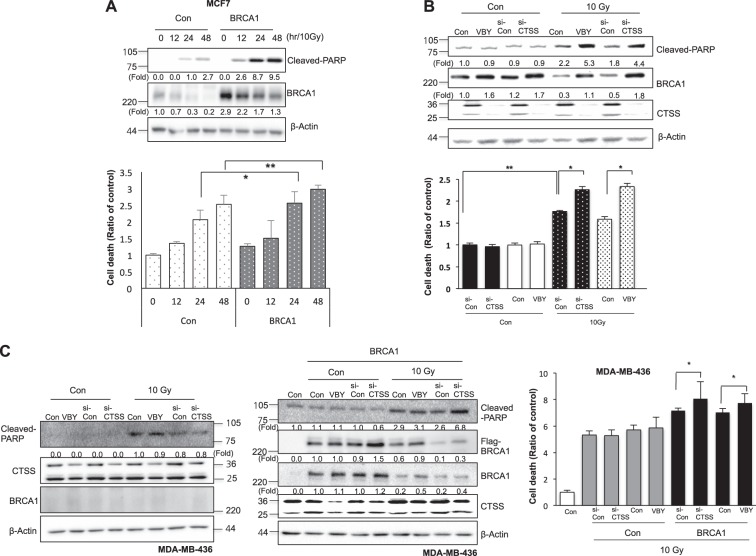
Inhibition of IR-mediated cell death by CTSS. **a** MCF7 cells were transfected with a control or WT-BRCA1. Western blots of cleaved-PARP and BRCA1 expression were performed (upper). Cell death was evaluated by PI staining after 12, 24, and 48 h of 10 Gy IR (bottom). **b** MCF7 cells were transfected with si-CTSS or were treated with VBY-036 (10 μM). Western blots of cleaved-PARP and BRCA1 expression were performed (upper). Prevalence of cell death was evaluated by PI staining after 48 h of exposure to 10 Gy (bottom). **c** MDA-MB-436 cells with or without WT-BRCA1 were treated with si-CTSS or VBY-036 (10 μM). After 48 h of 10 Gy IR, Western blotting (left) or PI staining (right) was performed. Protein levels were quantified using Image J software, and data are expressed as the fold change relative to the negative control. The graphs depict the mean ± SD of PI-positive cells. *p < 0.05 and **p < 0.01 (ANOVA)

BRCA1 is reported to increase IR-mediated apoptosis [49], and the present results also suggest that BRCA1 over-expression increased IR-mediated PARP cleavage and cell death (Fig. 7a). CTSS inhibition by si-CTSS or the specific inhibitor VBY increased IR-mediated PARP cleavage accompanied by cell death with increased BRCA1 expression in the case of MCF7 cells with intact BRCA1 functions (Fig. 7b). MDA-MB-231 cells with wild-type BRCA1 showed similar effects (Supplementary Figure S4). However, in the case of BRCT-mutated cells with low BRCA1 expression, such as MDA-MB-436 cells, CTSS inhibition did not result in any additional increase of PARP cleavage or cell death induced by IR; however, when we transfected WT-BRCA1 into these cells, CTSS inhibition increased IR-mediated PARP cleavage and cell death (Fig. 7c).

### CTSS involved in breast cancer development

**Fig. 8:**
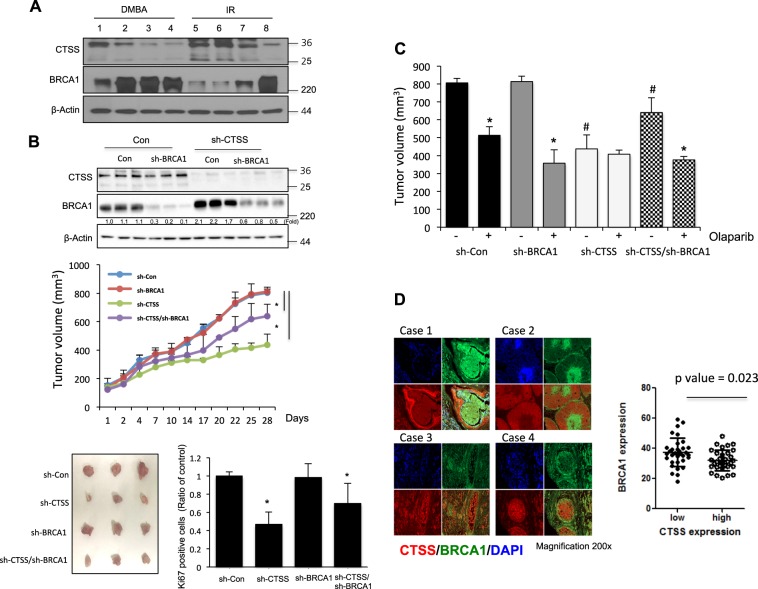
Delayed tumor growth by inhibition of CTSS with recovery of BRCA1 expression. **a** Western blotting (total 8 tissues, upper) or immunohistochemistry (IHC) (total 4 tissues, bottom) for BRCA1 and CTSS was performed using rat mammary tumor tissues. **b** Western blotting was performed using MBA-MD-231 cells stably transfected of sh-CTSS, sh-BRCA1, and sh-CTSS/sh-BRCA1 (double deletion of CTSS/BRCA1) (upper). Changes in the tumor volume in xenografted SCID mice (*n* = 5/group) after injection of MDA-MB231 cells (1 × 10^7^) were detected. Results are the means and standard deviations (*p < 0.05, Student’s *t*-test) (middle). Quantification of Ki67 positive cells after IHC analysis was performed (bottom) using image J software (NIH). *p < 0.05 (ANOVA). **c** Tumor growth measurement by treatment of oraparib in xenografted SCID mice (*n* = 5/group) at 28^th^ day of MDA-MB-231 cells injection. *p < 0.05 vs. corresponding olaparib untreated control group and ^#^*p* < 0.05 vs. olaparib untreated sh-control group (ANOVA). **d** IHC for BRCA1 and CTSS using human mammary cancer tissue microarray (*n* = 70), was performed using fluorescence-conjugated antibodies (CTSS: red; BRCA1: green). Photographs of four representative cancer tissues are presented. Quantification of BRCA1 positive and CTSS positive areas in each slide were analyzed using GraphPad Prism software 5.0

To elucidate the physiological role of CTSS in mammary tumorigenesis, we examined the relationship between CTSS and BRCA1 expression in spontaneously induced rat mammary tumors. Rat mammary tumors induced by DMBA or IR [42] were all malignant adenocarcinomas. The high CTSS-expressing mammary tumors (1, 5, 6, and 7) showed low BRCA1 expression, whereas the low CTSS-expressing mammary tumors (2, 3, 4, and 8) showed high BRCA1 expression (Fig. 8a and Supplementary Figure S5A). To elucidate the direct relationship between CTSS and BRCA1 in tumorigenesis, we examined tumor development after injection of sh-CTSS-, sh-BRCA1- or double sh-CTSS/sh-BRCA1-MDA-MB-231 cells using an SCID xenograft mouse model. sh-CTSS group showed the most effective tumor delay response, while sh-BRCA1 group showed similar tumor development with that of sh-Control group. sh-CTSS/sh-BRCA1 group showed less tumor delayed response than that of sh-CTSS alone xenografted group, suggesting antitumor effects by CTSS inhibition is occurred only in the presence of functional BRCA1. Immunohistochemistry data of Ki67 showed similar patterns of tumor growth data (Fig. 8b). Treatment of olaparib, a PARP inhibitor showed effective tumor regression in all the mice except for sh-CTSS xenograft mice (sh-CTSS xenograft group did not show any additional tumor regression by olaparib treatment) (Fig. 8c and Supplementary Figure S5C). When a human breast cancer tissue slide that included 70 patient specimens were examined, BRCA1 and CTSS expression were evident in the breast tissues, and total expression levels of each protein did not show an inverse correlation, in contrast to the rat mammary tumors. However, even though BRCA1 expression was not co-localized with CTSS (co-localization factor *r*^2^ = 0.31 by ANOVA analysis, Supplementary Figure S6), BRCA1 level was evidently low in the high CTSS expressed region within the slide (*p* = 0.023, Fig. 8d), suggesting that high CTSS expressing cells might have low BRCA1 expression.

## Discussion

BRCA1 mutations account for a significant proportion of familial breast and ovarian cancers. In addition, reduced BRCA1 is associated with sporadic cancers in these tissues; loss of BRCA1 function has been implicated in the development of breast and ovarian cancer. Therefore, maintaining BRCA1 functions may be important for inhibiting breast and ovarian cancers. Although it has been shown that BRCA1 turnover is achieved by ubiquitin-dependent proteolysis, the factors that regulate BRCA1 stability are largely unknown. In this study, we showed that BRCA1 stability is regulated by CTSS and that cleavage of the BRCT domain of BRCA1 by CTSS is important for the ubiquitin-mediated degradation of BRCA1, implicating CTSS inhibition as a therapeutic target for maintaining intact BRCA1 function.

Our previous study showed that CTSS was over-expressed in IR-induced rat mammary tumors [43]. Because CTSS is a protease, we screened for target proteins with a focus on DNA damage response factors. CTSS over-expression affected BRCA1 protein stability, but other DNA damage proteins such as ATM, DNA-PKcs, Ku80, and Cyclin D1 were not affected. Without altering the mRNA level of BRCA1, CTSS cleaved the BRCT-domain of BRCA1, resulting in BRCA1 ubiquitination. The results of another study also showed that cytosolic proteases such as cathepsins may regulate BRCA1 degradation [51]. Although that study’s authors did not provide clear evidence, their data suggested that BRCA1 is normally degraded by lysosomal proteases and that in the absence of lysosomal degradation, nuclear accumulation of BRCA1 occurs. They also showed that the amino terminus of BRCA1 harbors a degron sequence that is necessary for conferring BRCA1 degradation [50]. In our study, DNA damage-inducing factors such as IR increased and activated CTSS, which cleaved the BRCT domain of BRCA1. However, the RING domain was not essential for CTSS-mediated degradation and ubiquitination of BRCA1, suggesting that RING domain-independent BRCA1 degradation may also be an important determinant of BRCA1 stability. Indeed, a BRCT-domain deletion mutant exhibited decreased BRCA1 function such as promoter activity of *gadd45* and downstream pathways whereas RING-domain mutants did not affect *gadd45* promoter activity, suggesting that the BRCT domain is more important than the RING domain for BRCA1 functions. Indeed, CTSS did not affect BRCA1 degradation in cells with inherited BRCT-domain mutation and low BRCA1 expression. Moreover, CTSS inhibited the induction of HR repair and increased γH2AX expression, as well as inhibited the cell death in response to IR, and may be involved in BRCA1-deficiency mediated genomic instability and anti-apoptosis, suggesting that CTSS inhibits intact BRCA1 roles. Although one related paper suggested that mutations in the BRCT domain of BRCA1 frequently create protein products that are unable to fold and are subject to protease-mediated degradation, and that the inherent partial function of a BRCT domain-mutated BRCA1 protein can contribute to HR, our results are the first report of BRCT domain-dependent degradation of BRCA1 by CTSS. Regulation of BRCA1 degradation was specific for CTSS in that knockdown of CTSL or CTSB did not restore BRCA1 stability. We do not know the exact mechanisms that regulate CTSS-specific BRCA1 degradation, but one possible explanation is pH: CTSS is the only pH-dependent protease and IR affects the pH of the cells [52, 53], which may affect the tumor microenvironment and activate BRCA1 degradation.

Tissues of mammary tumors that were spontaneously induced by IR or DMBA and had different BRCA1 expression levels showed an inverse correlation between BRCA1 and CTSS, suggesting that CTSS regulates BRCA1 stability. Indeed, CTSS depletion restored BRCA1 expression and delayed tumor growth in a tumor xenograft model, which was not occurred in both CTSS and BRCA1 double deficiency tumors, suggesting the role of CTSS in cancer cells with intact BRCA1 function. To determine these relationships in human breast cancers, we used human breast cancer tissue arrays and found inverse correlation of expression patterns of CTSS and BRCA1 within each slide, suggesting possibly targeting CTSS to BRCA1 even in human breast cancer tissues.

Pharmacological inhibition of CTSS significantly reduced experimental breast cancer metastasis and tumor development [54], suggesting its potential as a therapeutic target for this disease. In this study, we also demonstrated that pharmacological inhibition of CTSS suppresses BRCA1 degradation and restores BRCA1 function, providing supporting evidence for CTSS as a potential target for anticancer therapy.

## Materials and Methods

### Cell culture

MDA-MB-231 (wild-type BRCA1), MDA-MB-436 (BRCA1-BRCT domain mutated, low expression of BRCA1), and MCF7 (wild-type BRCA1) cells were cultured in Dulbecco’s minimal essential medium (DMEM) (Gibco, Gaithersburg, MD, USA), supplemented with heat-inactivated 10% fetal bovine serum (FBS; Gibco), 0.1 mM nonessential amino acids, glutamine, HEPES, and antibiotics at 37 °C in a 5% CO_2_ humidified incubator.

### Cell transfection

Plasmids were generated by reverse PCR using human breast cDNA as template (Cosmogenetech, Seoul, Korea) and cloned into pcDNA3.1 and p3XFLAG-myc-CMV, a mammalian expression vector. CTSS and BRCA1 expression were suppressed using specific si-RNAs. si-CTSS, si-BRCA1 or si-Control(used as negative control) were purchased from Bioneer (Daejeon, Korea). CTSS sh-RNA plasmid (sc-29941-SH), sh-BRCA1 (sc-29219-SH), and sh-control plasmid (sc-10808) were from Santa Cruz Biotechnology (Dallas, TX, USA), and sh-RNA plasmid (sc-108080) were transfected with the lentiviruses in the presence of polybrene (sc-134220). CTSS CRISPR/Cas9 knockout (KO) plasmid (sc-417407) and control CRISPR/Cas9 control (Con) plasmid (sc-418922) were from Santa Cruz Biotechnologies. For transfection, cells were seeded in culture dishes and transfection was performed after 24 h using Opti-MEM media (Invitrogen, Carlsbad, CA, USA) containing Lipofectamine 2000 reagent (Invitrogen).

### CTSS activity

Cysteine protease activity was measured with assay kits for CTSS (ab65307). Cathepsin S activity assay kit was purchased from Abcam (Cambridge, MA, USA). Isolated cells were frozen and sonicated before being adding to ice-cold buffer from the assay kit. The enzymatic assay itself was conducted according to the manufacturer’s instructions.

### Cellular fractionation

Cellular fractionation was performed using a subcellular protein fractionation kit (Cat No.78841) purchased from Thermo Fisher Scientific (Waltham, MA, USA). Cultured cells were washed twice with ice-cold PBS and harvested with a scraper. Cell pellets were resuspended in cytoplasmic extraction buffer and incubated at 4 °C for 10 min. Samples were agitated every 5 min and then centrifuged at 12,000 rpm for 30 s to collect the cytoplasmic fractions. Pellets were resuspended, incubated in nuclear extraction buffer for 30 min, and centrifuged at 12,000 rpm for 20 min to obtain the nuclear fraction.

### *Gadd45* promoter assay

*Gadd4l5* WT-luc plasmid was purchased from Addgene (Cambridge, MA, USA). *Gadd45* promoter activity was measured using a luciferase assay system kit (Cat No. E4030) was purchased from Promega (Madison, WI, USA). Protein was quantitated using protein assay reagent (Cat No. #500–0006) was purchased from Bio-Rad (Hercules, CA, USA).

### Treatment of CTSS inhibitors

Cells were treated with the CTSS-specific inhibitor VBY-036 and the pan-cathepsin family inhibitor E-64 at final concentrations of 10 μM and 100 μM, respectively. VBY-036 was provided by Virobay (Palo Alto, CA, USA), and E-64 was purchased from Sigma Aldrich (St. Louis, MO, USA). Both agents were dissolved in DMSO before use.

### Immunoblotting and immunoprecipitation

Immunoblotting and immunoprecipitation were performed with antibodies against the following proteins: BRCA1 (sc-642), c-Myc (sc-64), p53 (sc-126), p21 (sc-6246), cyclin B1 (sc-245), lamin B (sc-6216), β-actin (sc-47778), and ubiquitin (sc-8017) were purchased from Santa Cruz Biotechnology. p-BRCA1 (9009 s), cleaved PARP (9541 s), HA- tag (#2367), γH2AX (5438 s), p-ATM (4526 s) were purchased from Cell Signaling. CTSS (ab92780), ATM (ab17995), GAPDH (ab9485) were purchased from Abcam. Antibodies of Flag (#F3165) and V5 (P/N 460705) were purchased from Sigma Aldrich and Invitrogen, respectively.

### Irradiation

Cells were plated in 60-mm dishes in culture medium and incubated at 37 °C in a humidified 5% CO_2_ atmosphere until 70–80% confluent. Cells were then exposed to γ-rays with a ^137^Cs γ -ray source (Elan 3000, Atomic Energy of Canada, Ontario, Canada) at a dose rate of 3.81 Gy/min.

### Flow cytometry

Cells were cultured, harvested, and fixed in 70% ethanol (1 × 10^6^ cells/sample) for 30 min at 4 °C. The cells were then washed twice with phosphate-buffered saline (PBS) and incubated in the dark for 10 min at 37 °C in PBS containing 10 μg/ml propidium iodide (Sigma Aldrich) and 10 μg/ml RNase A (Sigma Aldrich). Flow cytometric analysis was performed using a FACScan flow cytometer (Becton Dickinson, Franklin Lakes, NJ, USA).

### Immunofluorescence analysis

Cells were fixed with 2% paraformaldehyde, permeabilized with 0.1% Triton X-100 in PBS, washed three times with PBS, and incubated with anti-BRCA1 and anti-V5 antibodies diluted 1:200 in PBS with 5% FBS for 1 h at room temperature in a humidified chamber. Excess antibody was removed by washing the coverslips three times with PBS before incubation with fluorescein isothiocyanate-conjugated secondary antibody (Dako, Glostrup, Denmark) at a 1:200 dilution in PBS with 5% FBS for 4 h. After they were washed three times with PBS, cover slips were mounted onto microscope slides using mounting reagent (Southernbiotech, Birmingham, AL, USA). The slides were analyzed using an apotome laser-scanning microscope (Carl Zeiss, Oberkohen, German).

### Spontaneous mammary tumor development and histological examination

Spontaneous mammary tumors were induced in female Sprague-Dawley (SD) rats by oral administration of DMBA (15 mg/rat, Sigma Aldrich) or whole-body radiation (1.5 Gy once a week, total five times) at a rate of 3.81 Gy/min. Rats were autopsied under ether anesthesia 26 weeks after DMBA treatment and 40 weeks after the completion of radiation treatment. Collected tumors were fixed in 10% neutral buffered formalin, and paraffin-embedded sections were routinely prepared and stained with hematoxylin and eosin (H&E) for histological evaluation. The grading of rat breast carcinomas parallels efforts with human breast carcinomas to establish a basis for estimating virulence and thus the probable prognosis. These studies were conducted under guidelines for the use and care of laboratory animals and were approved by the Institutional Animal Care and Use Committee of the Korean Institute of Radiological and Medical Sciences.

### Immunohistochemistry

Immunohistochemistry was performed with the following antibodies: BRCA1 (ab16780, Abcam) or CTSS (sc-271619, Santa Cruz Biotechnology) and mouse anti-Ki-67 (M7248, Dako). Human breast cancer tissue slides were purchased from US Biomax. For antigen retrieval, slides were placed in citric acid buffer (pH 6.0) and heated at 100 °C for 10 min. Slides were incubated overnight at 4 °C with antibodies. Quantification of images was measured with image analyzer (Image J, NIH, Bethesda, MD, USA). All statistical analyses of images were performed using GraphPad Prism software 5.0 (GraphPad Software, San Diego, CA, USA).

### In vitro protein-binding assay

Pull-down assays were performed by incubating GST-BRCA1 fusion proteins loaded on glutathione-sepharose beads with cellular lysates in binding buffer for 18 h at 4 °C. The beads were washed extensively, resuspended in sample buffer, and analyzed by SDS-PAGE and western blotting with the indicated antibodies.

### Determination of HR or NHEJ activity

The DNA repair assay was performed as described previously [44]. Briefly, MCF7 cells stably expressing pimDR-GFP or pimEJ5-GFP (Addgene) were transfected with 4 μg of I-SceI (pCB-Asce) with 20 μl of Lipofectamine 2000 (Invitrogen) in 1 ml of OptiMEM (Gibco) with 4 μg of Mock vector, V5-CTSS, or Flag-BRCA1. The medium was changed 6 h after transfection. The cells were incubated for 72 h, and the percentage of GFP-positive cells were determined by fluorescence-activated cell sorting (FACS) analysis.

### Cycloheximide chase assay

BRCA1 stability was measured in the presence of cycloheximide (CHX, Sigma Aldrich) after CTSS over-expression or CTSS knockdown. At 24 h after transfection, the cells were split into multiple dishes, CHX was added (100 μg/ml), and the cells were harvested at the indicated times.

### Tumor xenograft experiments using SCID mice and immunohistochemistry

Cells were transfected with sh-CTSS, sh-BRCA1, or control shRNA lentiviral particles according to the manufacturer’s protocol. 5 × 10^5^ MDA-MB-231 cells were plated in a 6-well plate alongside 2 ml complete optimal medium with 5 µg/ml polybrene media mixture and 2 µl shRNA lentiviral particles per well. The transfected cells were selected with puromycin treatment (5 μg/ml for 2 days). Six-week-old female SCID (Envigo, Huntingdon, England) mice were used. 1 × 10^7^ cells of control MDA-MB-231 and stably sh-CTSS transfected MDA-MD-231 were injected subcutaneously. NOD/SCID mice were treated with 50 mg/kg olaparib (AZD2281, Selleckchem, Huston, TX, USA) daily, administered by i.p. injection, for 28 consecutive days. Tumor growth was monitored three times per week to assess treatment efficacy. Tumors were measured and monitored from 1 week after MDA-MB-231 cell were injected. At least five mice were used for each group. Double knockout cells with BRCA1 and CTSS (sh-BRCA1/sh-CTSS) were generated by additional transfection of sh-BRCA1 to sh-CTSS cells.

### Statistical analysis

Statistical significance was determined by ANOVA or Student’s *t*-test. Differences were considered significant if the *p* value was <0.05.

## Electronic supplementary material

Supplementary figure 1, Supplementary figure 2, Supplementary figure 3, Supplementary figure 4, Supplementary figure 5, Supplementary figure 6

Supplementary information

## References

[1] Scully R, Livingston DM (2000). In search of the tumour-suppressor functions of BRCA1 and BRCA2. Nature.

[2] Mullan P, Quinn J, Harkin D (2006). The role of BRCA1 in transcriptional regulation and cell cycle control. Oncogene.

[3] Zhu Q, Pao GM, Huynh AM, Suh H, Tonnu N, Nederlof PM (2011). BRCA1 tumour suppression occurs via heterochromatin-mediated silencing. Nature.

[4] Scully R, Chen J, Ochs RL, Keegan K, Hoekstra M, Feunteun J (1997). Dynamic changes of BRCA1 subnuclear location and phosphorylation state are initiated by DNA damage. Cell.

[5] Moynahan ME, Chiu JW, Koller BH, Jasin M (1999). Brca1 controls homology-directed DNA repair. Mol Cell.

[6] Snouwaert JN, Gowen LC, Latour AM, Mohn AR, Xiao A, DiBiase L (1999). BRCA1 deficient embryonic stem cells display a decreased homologous recombination frequency and an increased frequency of non-homologous recombination that is corrected by expression of a brca1 transgene. Oncogene.

[7] Korlimarla A, Prabhu JS, Remacle J, Rajarajan S, Raja U, Anupama C (2016). Identification of BRCA1 deficiency using multi-analyte estimation of BRCA1 and its repressors in FFPE tumor samples from patients with triple negative breast cancer. PLoS One.

[8] Silver DP, Livingston DM (2012). Mechanisms of BRCA1 tumor suppression. Cancer Discov.

[9] Ouchi T, Monteiro AN, August A, Aaronson SA, Hanafusa H (1998). BRCA1 regulates p53-dependent gene expression. Proc Natl Acad Sci USA.

[10] Mallery DL, Vandenberg CJ, Hiom K (2002). Activation of the E3 ligase function of the BRCA1/BARD1 complex by polyubiquitin chains. EMBO J.

[11] Wu W, Sato K, Koike A, Nishikawa H, Koizumi H, Venkitaraman AR (2010). HERC2 is an E3 ligase that targets BRCA1 for degradation. Cancer Res.

[12] Lu Y, Li J, Cheng D, Parameswaran B, Zhang S, Jiang Z (2012). The F-box protein FBXO44 mediates BRCA1 ubiquitination and degradation. J Bio Chem.

[13] Lorick KL, Jensen JP, Fang S, Ong AM, Hatakeyama S, Weissman AM (1999). RING fingers mediate ubiquitin-conjugating enzyme (E2)-dependent ubiquitination. Proc Natl Acad Sci USA.

[14] Brzovic PS, Rajagopal P, Hoyt DW, King MC, Klevit RE (2001). Structure of a BRCA1–BARD1 heterodimeric RING–RING complex. Nat Struct Biol.

[15] Hashizume R, Fukuda M, Maeda I, Nishikawa H, Oyake D, Yabuki Y (2001). The RING heterodimer BRCA1-BARD1 is a ubiquitin ligase inactivated by a breast cancer-derived mutation. J Biol Chem.

[16] Ruffner H, Joazeiro CA, Hemmati D, Hunter T, Verma IM (2001). Cancer-predisposing mutations within the RING domain of BRCA1: loss of ubiquitin protein ligase activity and protection from radiation hypersensitivity. Proc Natl Acad Sci USA.

[17] Baer R, Ludwig T (2002). The BRCA1/BARD1 heterodimer, a tumor suppressor complex with ubiquitin E3 ligase activity. Curr Opin Genet Dev.

[18] Chen A, Kleiman FE, Manley JL, Ouchi T, Pan ZQ (2002). Autoubiquitination of the BRCA1· BARD1 RING ubiquitin ligase. J Biol Chem.

[19] Xia Y, Pao GM, Chen HW, Verma IM, Hunter T (2003). Enhancement of BRCA1 E3 ubiquitin ligase activity through direct interaction with the BARD1 protein. J Biol Chem.

[20] Sato K, Hayami R, Wu W, Nishikawa T, Nishikawa H, Okuda Y (2004). Nucleophosmin/B23 is a candidate substrate for the BRCA1-BARD1 ubiquitin ligase. J Biol Chem.

[21] Starita LM, Machida Y, Sankaran S, Elias JE, Griffin K, Schlegel BP (2004). BRCA1-dependent ubiquitination of γ-tubulin regulates centrosome number. Mol Cell Biol.

[22] Starita LM, Horwitz AA, Keogh MC, Ishioka C, Parvin JD, Chiba N (2005). BRCA1/BARD1 ubiquitinate phosphorylated RNA polymerase II. J Biol Chem.

[23] Kleiman FE, Wu-Baer F, Fonseca D, Kaneko S, Baer R, Manley JL (2005). BRCA1/BARD1 inhibition of mRNA 3′ processing involves targeted degradation of RNA polymerase II. Genes Dev.

[24] Manke IA, Lowery DM, Nguyen A, Yaffe MB (2003). BRCT repeats as phosphopeptide-binding modules involved in protein targeting. Science.

[25] Rodriguez M, Yu X, Chen J, Songyang Z (2003). Phosphopeptide binding specificities of BRCA1 COOH-terminal (BRCT) domains. J Biol Chem.

[26] Yu X, Chini CCS, He M, Mer G, Chen J (2003). The BRCT domain is a phospho-protein binding domain. Science.

[27] Glover JM, Williams RS, Lee MS (2004). Interactions between BRCT repeats and phosphoproteins: tangled up in two. Trends Biochem Sci.

[28] Glover JM (2006). Insights into the molecular basis of human hereditary breast cancer from studies of the BRCA1 BRCT domain. Fam Cancer.

[29] Harper JW, Elledge SJ (2007). The DNA damage response: ten years after. Mol Cell.

[30] Zhou BBS, Elledge SJ (2000). The DNA damage response: putting checkpoints in perspective. Nature.

[31] Deng CX (2006). BRCA1: cell cycle checkpoint, genetic instability, DNA damage response and cancer evolution. Nucleic Acids Res.

[32] Huen MS, Sy SM, Chen J (2010). BRCA1 and its toolbox for the maintenance of genome integrity. Nat Rev Mol Cell Biol.

[33] Powell SN, Kachnic LA (2003). Roles of BRCA1 and BRCA2 in homologous recombination, DNA replication fidelity and the cellular response to ionizing radiation. Oncogene.

[34] Olson OC, Joyce JA (2015). Cysteine cathepsin proteases: regulators of cancer progression and therapeutic response. Nat Rev Cancer.

[35] Jedeszko C, Sloane BF (2004). Cysteine cathepsins in human cancer. Biol Chem.

[36] Joyce JA, Hanahan D (2004). Multiple roles for cysteine cathepsins in cancer. Cell Cycle.

[37] Flannery T, Gibson D, Mirakhur M, McQuaid S, Greenan C, Trimble A (2003). The clinical significance of cathepsin S expression in human astrocytomas. Am J Pathol.

[38] Kos J, Sekirnik A, Kopitar G, Cimerman N, Kayser K, Stremmer A (2001). Cathepsin S in tumours, regional lymph nodes and sera of patients with lung cancer: relation to prognosis. Br J Cancer.

[39] Rodriguez J, Vazquez J, Corte M, Lamelas M, Bongera M, Corte M (2004). Clinical significance of cathepsin D concentration in tumor cytosol of primary breast cancer. Int J Biol Markers.

[40] Turk V, Turk B, Guncar G, Turk D, Kos J (2002). Lysosomal cathepsins: structure, role in antigen processing and presentation, and cancer. Adv Enzym Regul.

[41] Small DM, Burden RE, Jaworski J, Hegarty SM, Spence S, Burrows JF (2013). Cathepsin S from both tumor and tumor‐associated cells promote cancer growth and neovascularization. Int J Cancer.

[42] Lee HJ, Lee YJ, Kang CM, Bae S, Jeoung D, Jang JJ (2008). Differential gene signatures in rat mammary tumors induced by DMBA and those induced by fractionated γ radiation. Radiat Res.

[43] Seo HR, Bae SW, Lee YS (2009). Radiation-induced cathepsin S is involved in radioresistance. Int J Cancer.

[44] Choudhury AD, Xu H, Baer R (2004). Ubiquitination and proteasomal degradation of the BRCA1 tumor suppressor is regulated during cell cycle progression. J Biol Chem.

[45] Jin S, Zhao H, Fan F, Blanck P, Fan W, Colchagie AB (2000). BRCA1 activation of the GADD45 promoter. Oncogene.

[46] Wu W, Koike A, Takeshita T, Ohta T (2008). The ubiquitin E3 ligase activity of BRCA1 and its biological functions. Cell Div.

[47] Shabbeer S, Omer D, Berneman D, Weitzman O, Alpaugh A, Pietraszkiewicz A (2013). BRCA1 targets G2/M cell cycle proteins for ubiquitination and proteasomal degradation. Oncogene.

[48] Choulika A, Perrin A, Dujon B, Nicolas JF (1995). Induction of homologous recombination in mammalian chromosomes by using the I-SceI system of Saccharomyces cerevisiae. Mol Cell Bio.

[49] Thangaraju M, Kaufmann SH, Couch FJ (2000). BRCA1 facilitates stress-induced apoptosis in breast and ovarian cancer cell lines. J Biol Chem.

[50] Blagosklonny MV, An WG, Melillo G, Nguyen P, Trepel JB, Neckers LM (1999). Regulation of BRCA1 by protein degradation. Oncogene.

[51] Turk V, Stoka V, Vasiljeva O, Renko M, Sun T, Turk B (2012). Cysteine cathepsins: from structure, function and regulation to new frontiers. Biochim Biophys Acta.

[52] Reisz JA, Bansal N, Qian J, Zhao W, Furdui CM (2014). Effects of ionizing radiation on biological molecules—mechanisms of damage and emerging methods of detection. Antioxid Redox Signal.

[53] Chang WSW, Wu HR, Yeh CT, Wu CW, Chang JY (2007). Lysosomal cysteine proteinase cathepsin S as a potential target for anti-cancer therapy. J Can Mol.

[54] Lieber MR, Gu J, Lu H, Shimazaki N, Tsai AG. Nonhomologous DNA end joining (NHEJ) and chromosomal translocations in humans. *Subcell Biochem* 2010;50:279–296. 10.1007/978-90-481-3471-7_14 PMC3079314 20012587

