# Supplementary material for: Regulating BRCA1 protein stability by cathepsin S-mediated ubiquitin degradation
Source: Cell Death Differ. 2018 Jul 13;26(5):812–25. doi: 10.1038/s41418-018-0153-0 (PMC6461859; doi:10.1038/s41418-018-0153-0)

Supplementary Figure S1

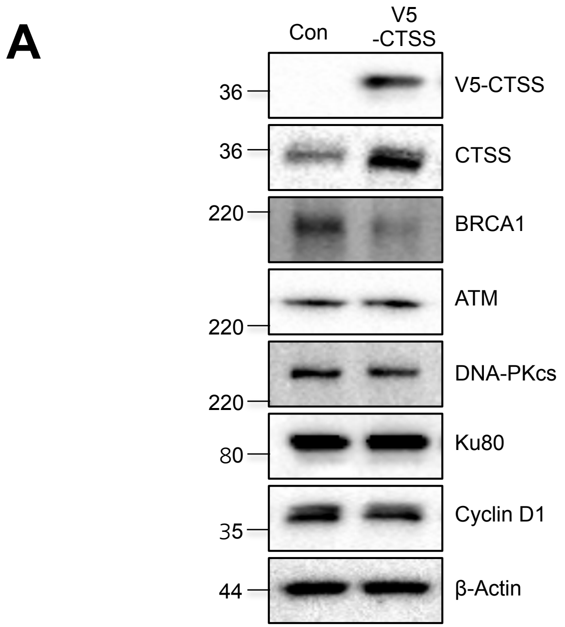

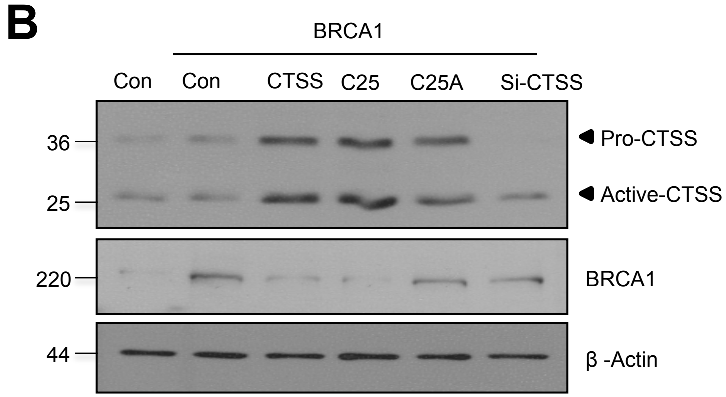

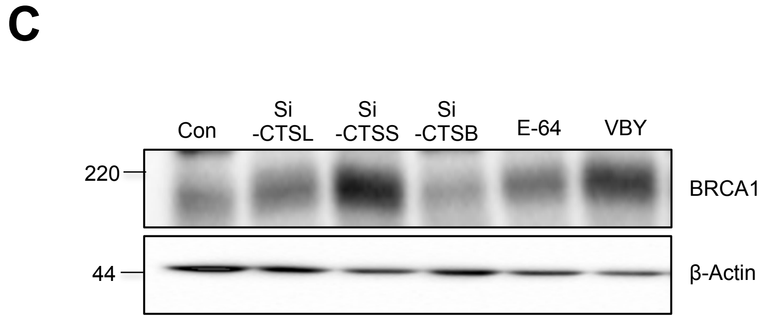

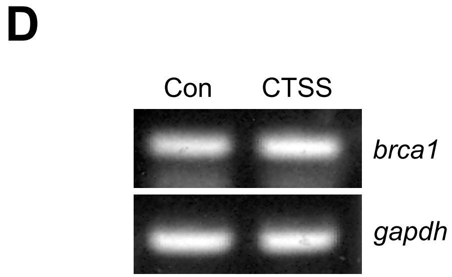

Supplementary Figure S2

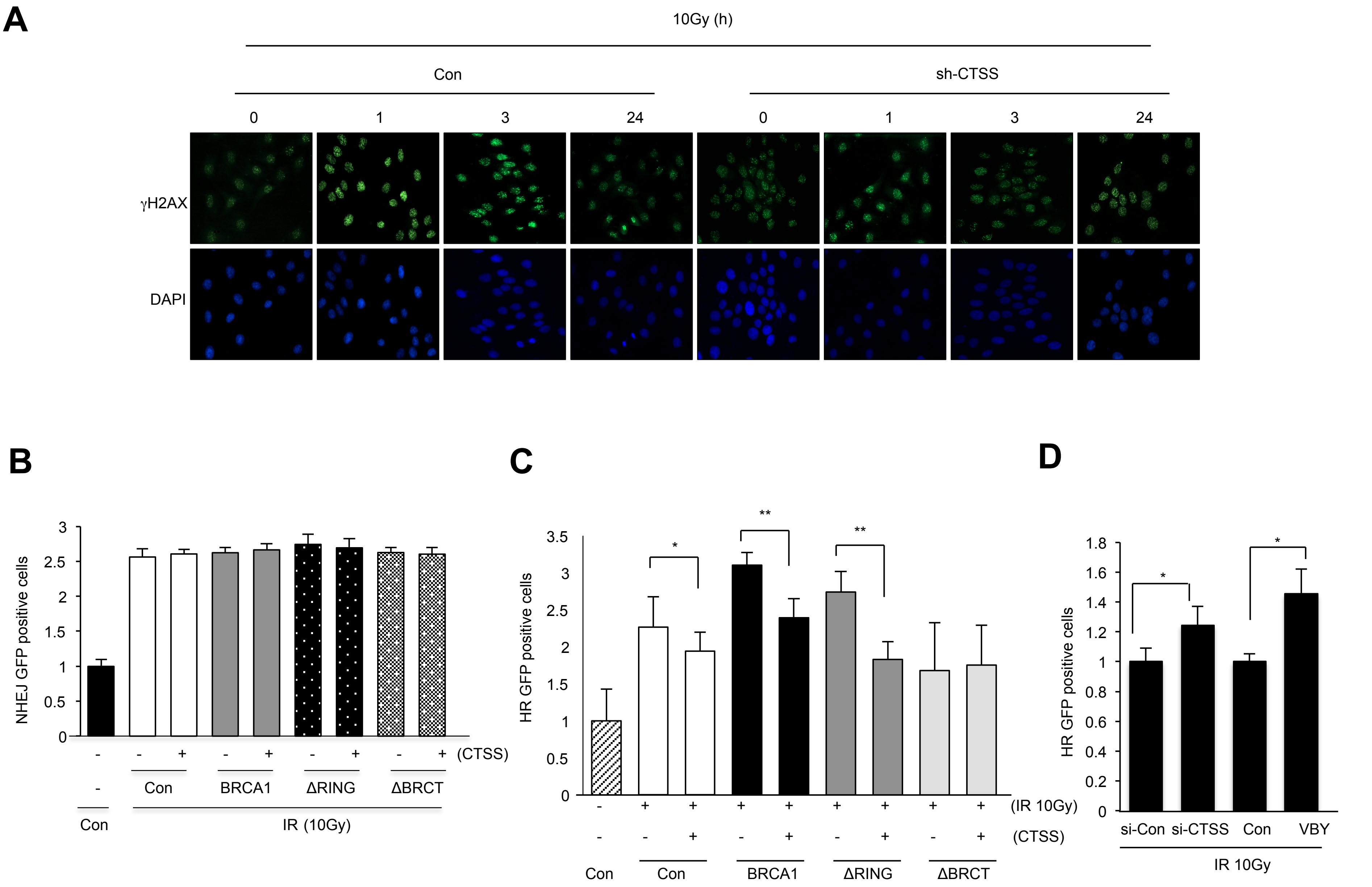

Supplementary Figure S3

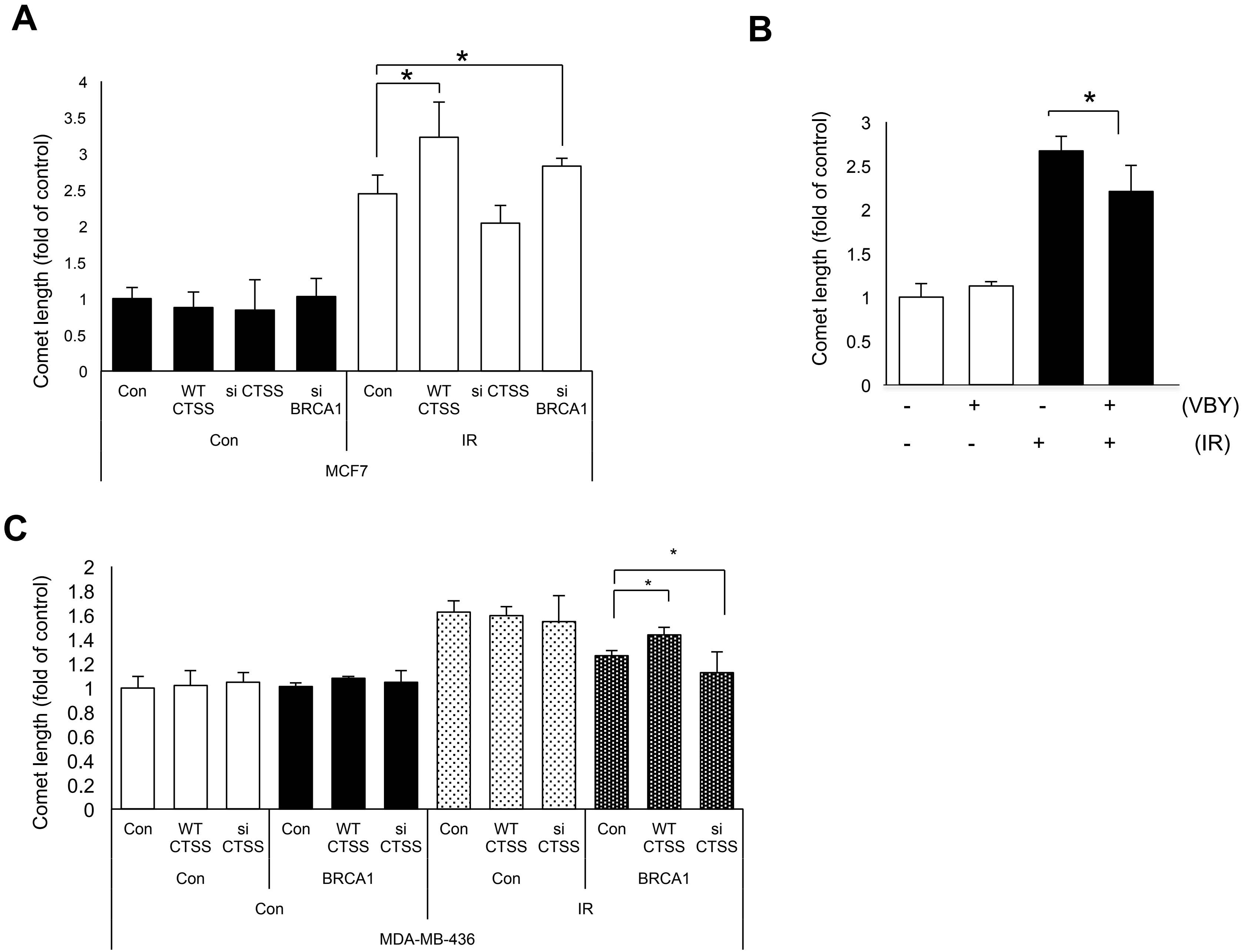

Supplementary Figure S4

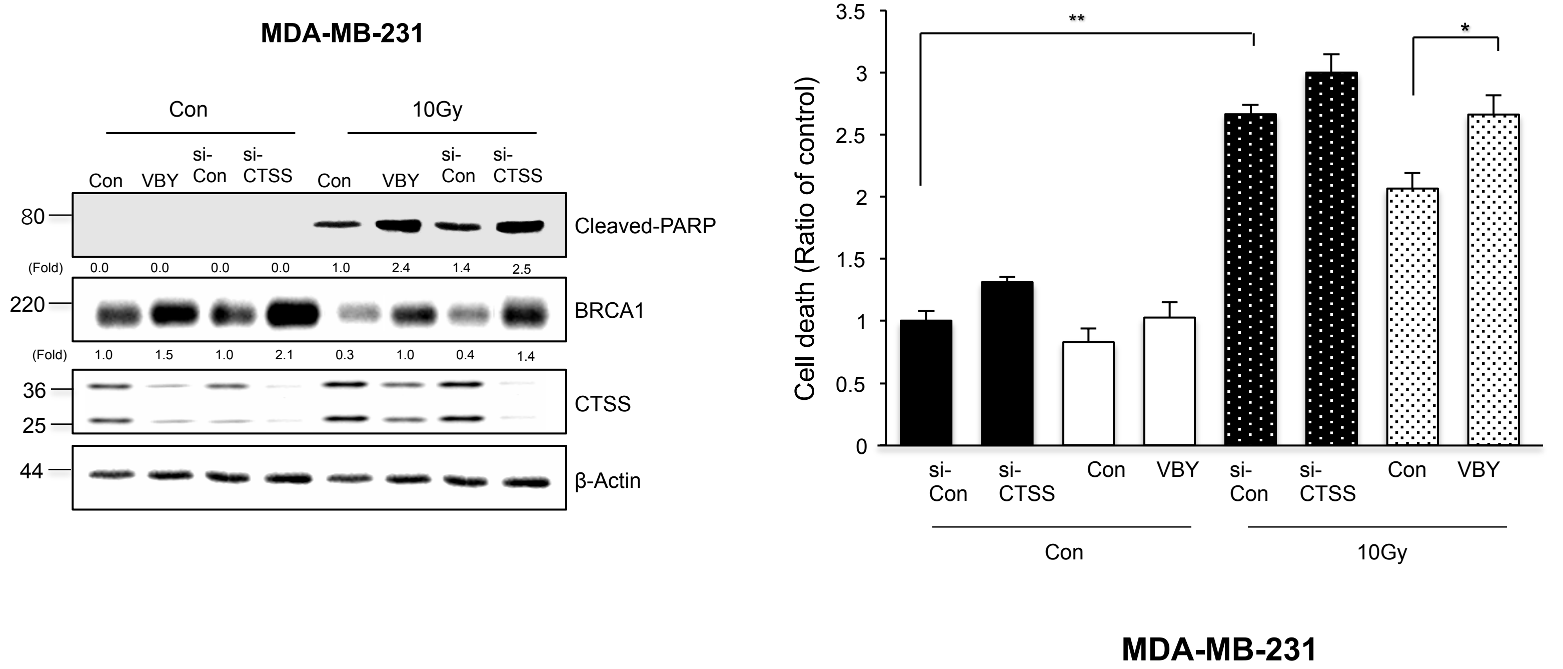

Supplementary Figure S5

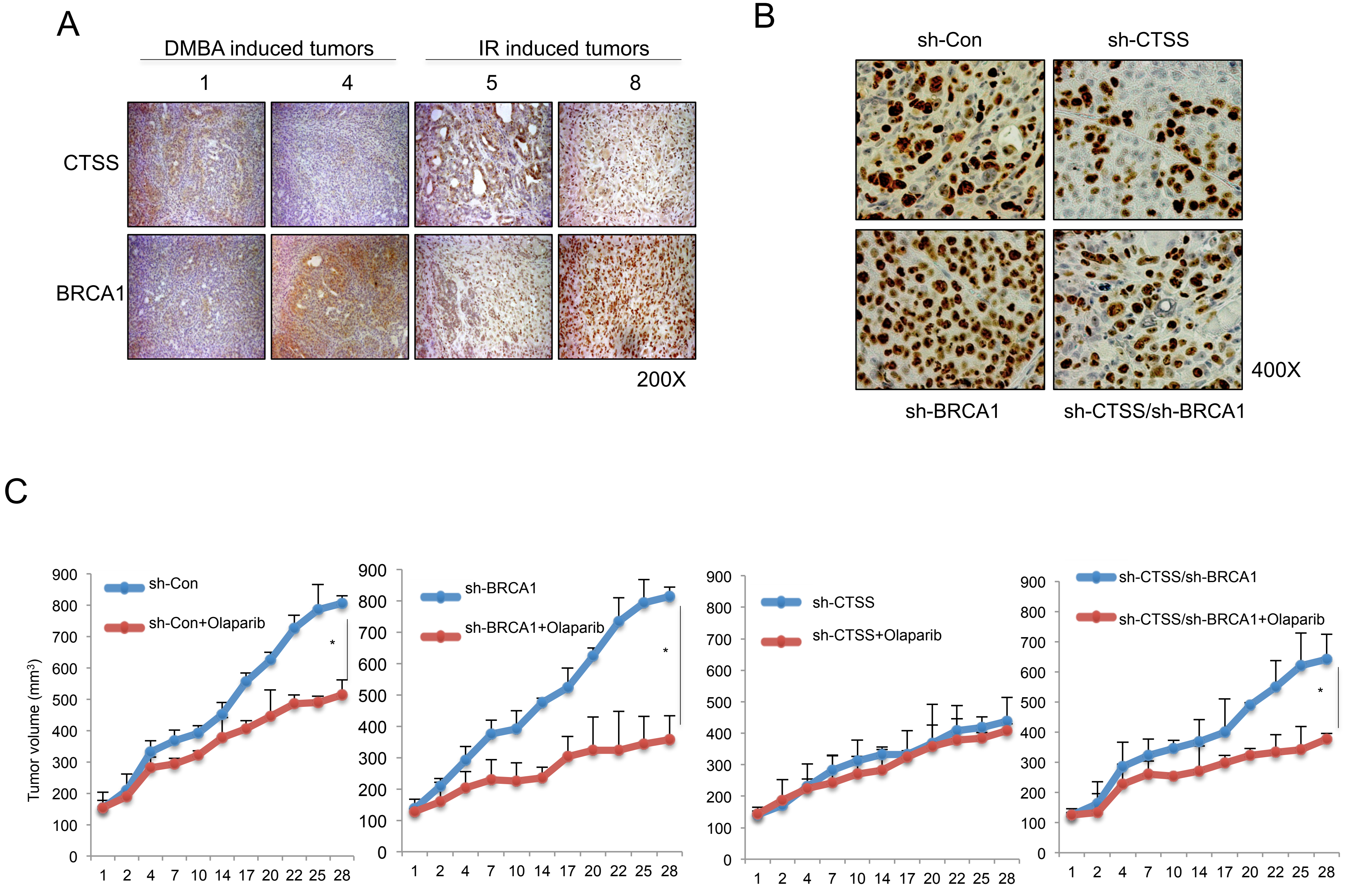

Supplementary Figure S6

A

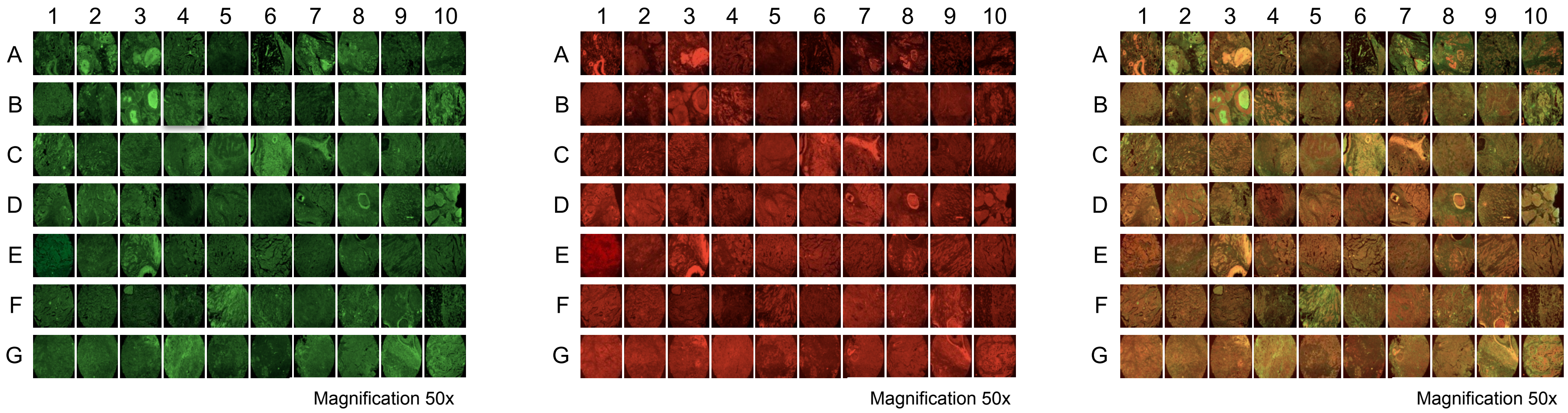

B

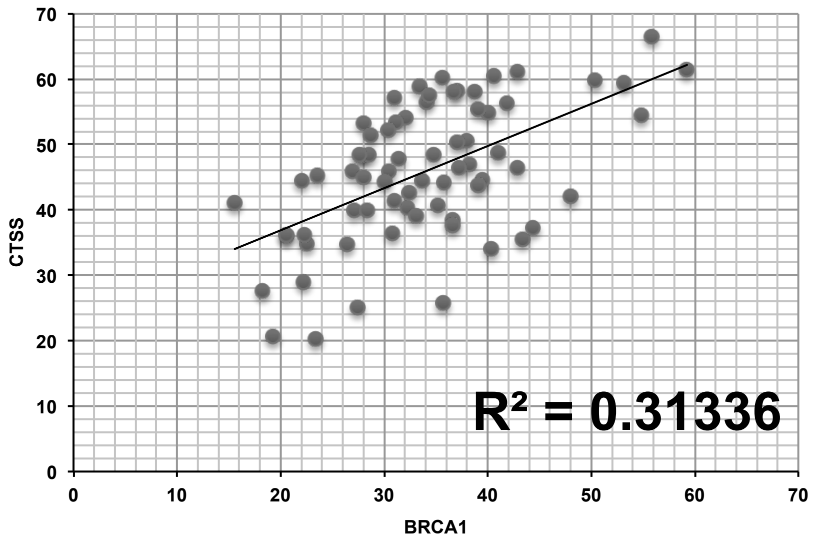

C

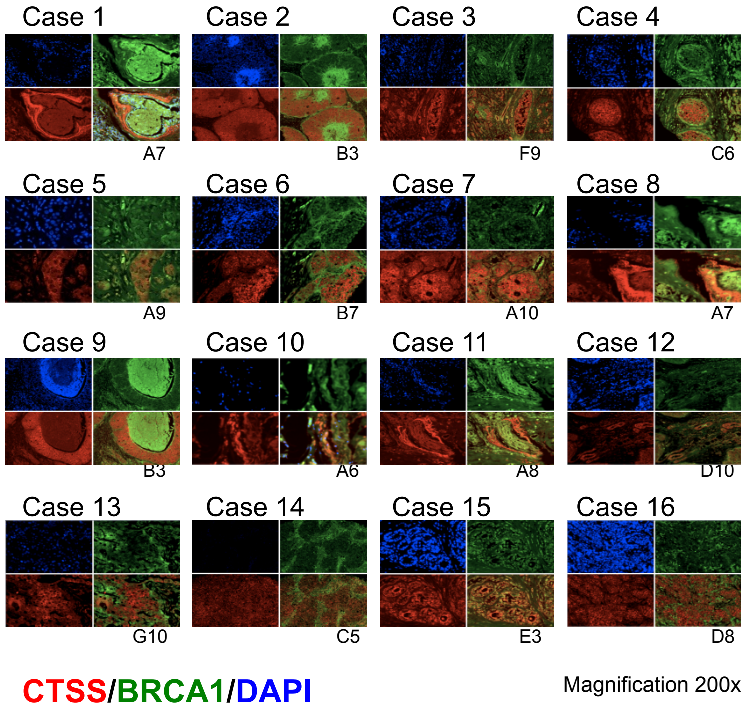

Supplement: Supplementary file 1 — Supplementary figure 1, Supplementary figure 2, Supplementary figure 3, Supplementary figure 4, Supplementary figure 5, Supplementary figure 6 [file 41418_2018_153_MOESM1_ESM.pdf]
