# Supplementary material for: Regulating BRCA1 protein stability by cathepsin S-mediated ubiquitin degradation
Source: Cell Death Differ. 2018 Jul 13;26(5):812–25. doi: 10.1038/s41418-018-0153-0 (PMC6461859; doi:10.1038/s41418-018-0153-0)
Supplement: Supplementary file 2 — Supplementary information [file 41418_2018_153_MOESM2_ESM.docx]

**Materials and Methods**

**Comet assay.**

The transfected cells were exposed to IR and subjected to a comet assay to detect DNA damage and repair at the single-cell level, using a commercially available assay system (Trevigen, Helgerman, MD, USA). To determine the level of DNA damage in breast cancer cells (MCF7, MDA-MB-231, MDA-MB-436) were treated with 10 Gy radiation subjected to a comet assay to detect DNA damage and repair at the level of single cells under neutral conditions. Briefly, after treatment, cells were harvested and mixed with low-melting-temperature agarose. After lysis, electrophoresis was performed at 1 V/cm and 15 mA for 40 min. Slides were stained with SYBG Green dye for 10 min. 250 randomly selected cells per sample were captured under a fluorescent microscope, and digital fluorescent images were obtained using AxioVision software (Cal Zeiss, Oberkohen, Germany). The relative length and intensity of SYBR Green–stained DNA tails to heads is proportional to the amount of DNA damage present in the individual nuclei and is measured by Olive tail moment using TriTek CometScore software (TriTek, Valencia, CA, USA).

**RNA extraction and RT-PCR analysis**

The MCF7 cells were seeded into 100cm dishesat a density of 2×10^5^ cells/well in 7 ml medium. The cells were transfected with brca1 for 24 h. Total RNA was isolated using TRIzol reagent. Oligo(dT)-primed RNA (1 μg) was reverse transcribed with SuperScript II reverse transcriptase according to the manufacturer's instructions. The obtained cDNA was used to determine the amount of brca1 and glyceraldehyde 3-phosphate dehydrogenase (GAPDH) mRNA using PCR with Taq DNA polymerase. GAPDH was used as an internal control. The primers that were used for amplification of the BRCA1 transcripts were as follows: BRCA1 forward, 5'-ACAGCTGTGTGGTGCTTCTGTG-3' and reverse, 5'-CATTGTCCTCTGTCCAGGCATC-3'; and GAPDH forward, 5′-GT CAT CCA TGA CAA CTT TGG-3′ and reverse, 5′-GA GCT TGA CAA AGT GGT CGT-3′.

Legends

Supplementary Figure S1

(A) Western blot analysis demonstrating expression levels of the DNA damage-related proteins in MCF7 cells transfected with WT-CTSS. (B) MDA-MB-231 cells were transfected with WT-CTSS, mutant type of CTSS (C25; the active site of Cys of CTSS, C25A, replaced the active site of Cys25 of CTSS to Ala) or RNAi and Western blotting was performed. (C) MCF7 cells were transfected with si-CTSS, si-CTSL, or si-CTSB or treated with E64 (cathepsin family inhibitor), VBY (CTSS specific inhibitor); then, Western blotting was performed. (D) mRNA expression levels of *brca1* and *gapdh* gene in MCF7 cells transfected with WT-CTSS.

Supplementary Figure S2

(A) Representative images for γ-H2AX foci in control or sh-CTSS transfected cells after 10 Gy IR. (B) MCF7-NHEJ cells were transfected by individual plasmids. After 10 Gy IR, the I-SecI expression construct was transfected at 24 h after transfection, and cells were incubated for additional 48 h. The numbers of GFP-positive cells were determined by fluorescence-activated cell sorting. The assays were repeated at least four times (n = 3), and the average relative numbers are presented as the mean ± SD. (C) After 10 Gy IR, the ratio of GFP+ cells transfected with sh-CTSS, BRCA1, ΔBRCT, or ΔRING expression in MCF7 cells that stably expressed EJ5-GFP were analyzed by FACS. (D) GFP+ cells after treatment of VBY-036 (10 μM) or sh-CTSS in 10 Gy irradiated MCF7 cells that stably expressed EJ5-GFP were analyzed by FACS (mean ± SD from three different experiments). *p<0.05 and **p<0.01 (ANOVA)

Supplementary Figure S3

(A) MCF7 cells were transfected with the indicated plasmids. Quantification of the percentage of nuclei with comet tails, which indicate DSBs. Tail length (in μm), indicating the extent of DNA fragmentation, measured for each cell showing a comet. (B) MCF7 cells were treated with VBY (cathepsin S specific inhibitor) for 24 h. Transfected cells were irradiated with 10 Gy. Two h later, comet assay was performed. (C) MDA-MB-436 (triple-negative breast cell line; BRCA1 mutation) cells were transfected with individual plasmids. The comet tail moments represent the averages of 250 cells in three independent experiments. **p<0.05* (ANOVA)

Supplementary Figure S4

MDA-MB-231 cells were transfected with si-CTSS or were treated with VBY-036 (10 μM). Western blots of cleaved-PARP and BRCA1 expression were performed (left). Prevalence of cell death was evaluated by PI staining after 48 h of exposure to 10 Gy (right). Protein levels were quantified using Image J software, and data are expressed as the fold change relative to the negative control. The graphs depict the mean ± SD of PI-positive cells. **p<0.05* and ***p<0.01* (ANOVA).

Supplementary Figure S5

(A) The representative images of immunohistochemistry (IHC) for BRCA1 and CTSS. IHC staining was performed using rat mammary tumor tissues. (B) Stably transfection of sh-CTSS, sh-BRCA1, and sh-CTSS/sh-BRCA1 (double deletion of CTSS/BRCA1) to MDA-MB-231 cells was performed. Immunohistochemistry images of Ki67 staining in xenografts generated from subcutaneous transplantation of transfected MDA-MB-231 cells into SCID mice. (C) Changes in the tumor volume in xenograft SCID mice (n = 5/group) with sh-control, sh-BRCA1, sh-CTSS and sh-BRCA1/CTSS (double deletion of CTSS/BRCA1) stably expressed MDA-MB231 cells (1 × 10^7^) with or without olaparib. Tumor size was measured twice weekly. Results are the means and standard deviations (**p<0.05*, Student’s t test).

Supplementary Figure S6

(A) CTSS and BRCA1 levels in a breast cancer tissue. Immunofluorescence of tissue microarrays slides (n=70). In all images colors are as follows: BRCA1 (green), CTSS (red), Merge (BRCA1 and CTSS) (yellow), and DAPI (blue). All images are 50x magnifications. (B) Scatter plots demonstrated the intensity of BRCA1 and CTSS staining in human breast cancer tissues (n=70) and co-localization factor was analyzed by ANOVA. (C) Representative images for comparison of BRCA1 (green) and CTSS (red). Images are 200x magnification.
